# Anti-biofilm activity of caffeine against uropathogenic *E. coli* is mediated by curli biogenesis

**DOI:** 10.1038/s41598-022-23647-2

**Published:** 2022-11-07

**Authors:** Bhawna Rathi, Surbhi Gupta, Parveen Kumar, Veerbhan Kesarwani, Rakesh Singh Dhanda, Sandeep Kumar Kushwaha, Manisha Yadav

**Affiliations:** 1 Dr. B. R. Ambedkar Center for Biomedical Research, University of Delhi (North Campus), New Delhi, 110007 India; 2 Department of Urology, University of Alabama at Birmingham, Birmingham, AL USA; 3 Hap BiosolutionsPvt. Ltd., Bhopal, 462042 India; 4 Celluleris AB, VentureLab, Scheelevägen 15, 223 70 Lund, Sweden; 5 DBT-National Institute of Animal Biotechnology (NIAB), Hyderabad, India; 6 Department of Clinical Sciences, Lund University, Malmö, Sweden

**Keywords:** Computational biology and bioinformatics, Drug discovery, Microbiology, Molecular biology, Urology

## Abstract

Biofilms are assemblages of sessile microorganisms that form an extracellular matrix around themselves and mediate attachment to surfaces. The major component of the extracellular matrix of Uropathogenic *E. coli* and other Enterobacteriaceae are curli fibers, making biofilms robust and resistant to antimicrobials. It is therefore imperative to screen antibiofilm compounds that can impair biofilm formation. In the present study, we investigated the curli-dependent antibiofilm activity of caffeine against UPEC strain CFT073 and commensal strain *E. coli* K-12MG1655.Caffeine significantly reduced the biofilm formation of both UPEC and *E. coli* K-12 by 86.58% and 91.80% respectively at 48 mM caffeine as determined by Crystal Violet assay. These results were further confirmed by fluorescence microscopy and Scanning Electron Microscope (SEM). Caffeine significantly reduced the cytotoxicity and survivability of UPEC. Molecular docking analysis revealed a strong interaction between caffeine and curli regulator protein (Csg D) of *E. coli*. The qRT-PCR data also showed significant downregulation in the expression of CsgBA and the CsgDEFG operon at both 24 mM and 48 mM caffeine. The findings revealed that caffeine could inhibit *E. coli* biofilm formation by regulating curli assembly and thus may be used as an alternative therapeutic strategy for the treatment of chronic *E. coli* biofilm-related infections.

## Introduction

A biofilm consists of a complex community of microorganisms that differ from their planktonic counterpart and allow bacteria to survive under extreme conditions such as nutrient depletion and desiccation^1,2^. Biofilms-associated infections (e.g., implant-associated infections, urethritis, vaginitis,cystic fibrosis) are generally chronic in which the treatment is quite challenging as cells are embedded in a matrix of extracellular polymeric substances (EPS), making them less susceptible to antimicrobial agents, drugs, and host immunity^3–5^. It is thought that the increased microbial resistance is a result of the different cellular metabolism of biofilm cells compared to planktonic cells, as well as the presence of EPS which acts as a diffusion-limiting barrier to antibiotics, thus limiting access to the deeper layers of a biofilm^6,7^. Hence, there is a compelling need to discover antibiotic-sparing anti-biofilm compounds to combat these biofilm-related infections.

Biofilms are formed by a wide variety of organisms like *Salmonella*, *Pseudomonas,* and *Escherichia coli (E.coli)* which add to the economic cost of the medical settings^8^.The primary structural element of matrix in *E. coli* biofilms is the curli protein, typically amyloid fibers, which acts in numerous capacities in a biofilm^9,10^. Curli is the primary protein responsible for establishing surface attachment and directing the biofilm's overall structure in enteric biofilms^10–12^. The curli fimbriae on the UPEC (uropathogenic *E. coli*) surface facilitate interbacterial interactions which give bacteria an advantage to invade deeper tissues^13,14^. These findings suggest that curli biogenesis may be considered as potential targets for developing therapeutics that can attenuate biofilm formation^15^.

Curli biogenesis is a complicated process that produces curli fibers when enteric bacteria are subjected to stress^16^. Curli production is regulated by the two (curli-specific gene) operons csgBAC and csgDEFG^17^. CsgD is the master regulator of curli biogenesis. It binds to both operons and is responsible for triggering transcription of the csgBACoperon^18^. CsgA (major subunit) and CsgB (minor subunit) are curli's structural elements which are produced when csg D binds to the promoter of csg BAC operon. CsgB is responsible for nucleating CsgA into the fiber across the outer membrane via Csg G secretion complex. Csg E and Csg F help in the secretion of CsgA and CsgB across the membrane. The role of CsgC is to prevent the polymerization of CsgA in periplasmic space as polymerization in the cell could lead to cell death^16,19,20^.

Recently, many natural compounds with antibacterial properties have been viewed as potential antibiofilm agents.Natural anti-microbial agents are always preferred over synthetic analogs in developing alternative therapies due to their better efficacy, accessibility, and less toxicity^2,21^. One such promising candidate is caffeine (1,3,7-trimethylxanthine),an alkaloid commonly found in coffee, tea, and other beverages^22^. Caffeine possesses an array of physiological effects on many microorganisms at sub-millimolar concentrations^23^. Caffeine has many health benefits, such as improving memory, decreasing fatigue, improving mental functioning and preventing and treating many diseases like premature infant breathing disorders, type 2 diabetes, Parkinson’s disease, and liver diseases^24–26^. Studies have reported that caffeine might be interfering in DNA synthesis and bacterial DNA repair pathways, resulting in the retardation of the growth of *E. coli*^27,28^.

Therefore, we investigated the antibiofilm properties of caffeine on Uropathogenic *E. coli* (CFT073, pyelonephritis strain) and commensal *E. coli* strain (K12 MG1655). We have identified its potential impact on *E. coli* biofilm formation by regulating curli biogenesis. This study might be helpful in developing strategies designed to attenuate *E. coli* biofilm formation and in treatment of chronic *E. coli* biofilm infections.

## Results

### Caffeine showed antibacterial properties against both CFT073 and K12 MG1655

**Figure 1:**
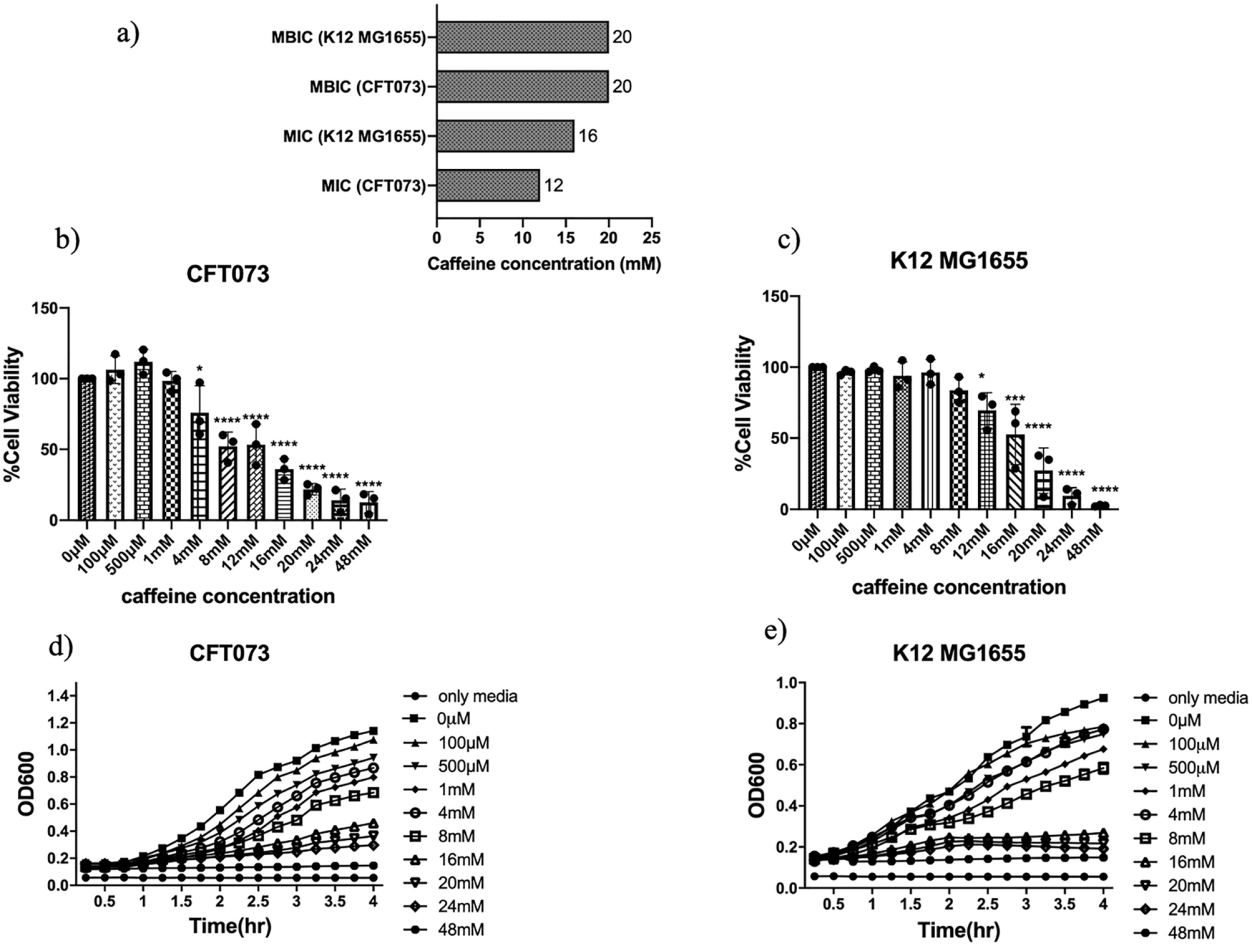
Effect of caffeine onantimicrobial activity,cell viability and growth kinetics profile of planktonic cells of CFT073 and K12MG1655: (**a**) MIC and MBIC determination of caffeine against CFT073 and K12 MG1655cellsin planktonic growth and their biofilms. (**b**) Effect of caffeine on the viability of CFT073 (**c**) Effect of caffeine on the viability of K12 MG1655. (**d**) Effect of caffeine on the growth of CFT073 bacteria grown in LB media supplemented with different concentrations of caffeine for 4 h. LB media without bacteria served as the negative control. CFT073 bacteria without caffeine served as the positive control. (**e**) Effect of caffeine on the growth of K12 MG1655 bacteria grown in LB media supplemented with different concentrations of caffeine for 4 h. LB media without bacteria served as the negative control. K12 MG1655 bacteria without caffeine served as the positive control. Optical Density at 600 nm was recorded every 15 min and the experiments were performed in triplicates. The data represents an average of three independent experiments presented as mean ± SD where *p < 0.05, **p < 0.01, ***p < 0.001,and ****p < 0.0001 indicates statistically significant difference relative to the untreated control (one-way ANOVA).

The antimicrobial activity of caffeine against most gram-negative and gram-positive bacteria was reported previously in the literature, where Minimum Inhibitory Concentration (MIC) against *E. coli* was found to be 10 mg/ml (51.49 mM)^29–31^. In the present study, the antibacterial activity of caffeine was evaluated by assessing the MIC and Minimum Biofilm Inhibitory Concentration (MBIC) against UPEC strain CFT073 and K12 MG1655. The MIC against CFT073 was found to be 12 mM and 16 mM against K12 MG1655 (Fig. 1a, Supplementary Figs. S1a,b and S2). However, the MBIC against both strains was found to be 20 mM (Fig. 1a, Supplementary Figs. S1c,d and S3). Supplementary Figs. S2 and S3 represents the photographic images of MIC and MBIC determination respectively. The results exhibited that caffeine had moderate antimicrobial activity against CFT073 and K12 MG1655.

Bacteria cell viability can be measured with MTT assay for evaluating the activity of antimicrobial agents against biofilms^32,33^. Therefore, the effect of caffeine on the viability of CFT073 and K12 MG1655 in the biofilm was checked by MTT assay. In CFT073 biofilms, the cell viability remained above 75% in the presence of 4 mM caffeine and then decreased significantly to only 12.73% at 48 mM caffeine (Fig. 1b). The cell viability in K12 MG1655 biofilms was found to be 83.69% at 8 mM caffeine, which was reduced to only 2.62% at 48 mM caffeine (Fig. 1c).

The growth curve was further investigated to identify the effects of caffeine on the growth of *E. coli*. In both strains, caffeine inhibited *E. coli* growth in a dose-dependent manner, even though the lower concentration (100 µM) of caffeine had no such effect (Fig. 1d,e). However, the growth of both strains was found to be completely ceased at 48 mM caffeine (Fig. 1d,e). These results suggest that caffeine might inhibit the biofilm formation ability of *E. coli*.

### Caffeine inhibited the biofilm forming capability of CFT073 and K12 MG1655

**Figure 2:**
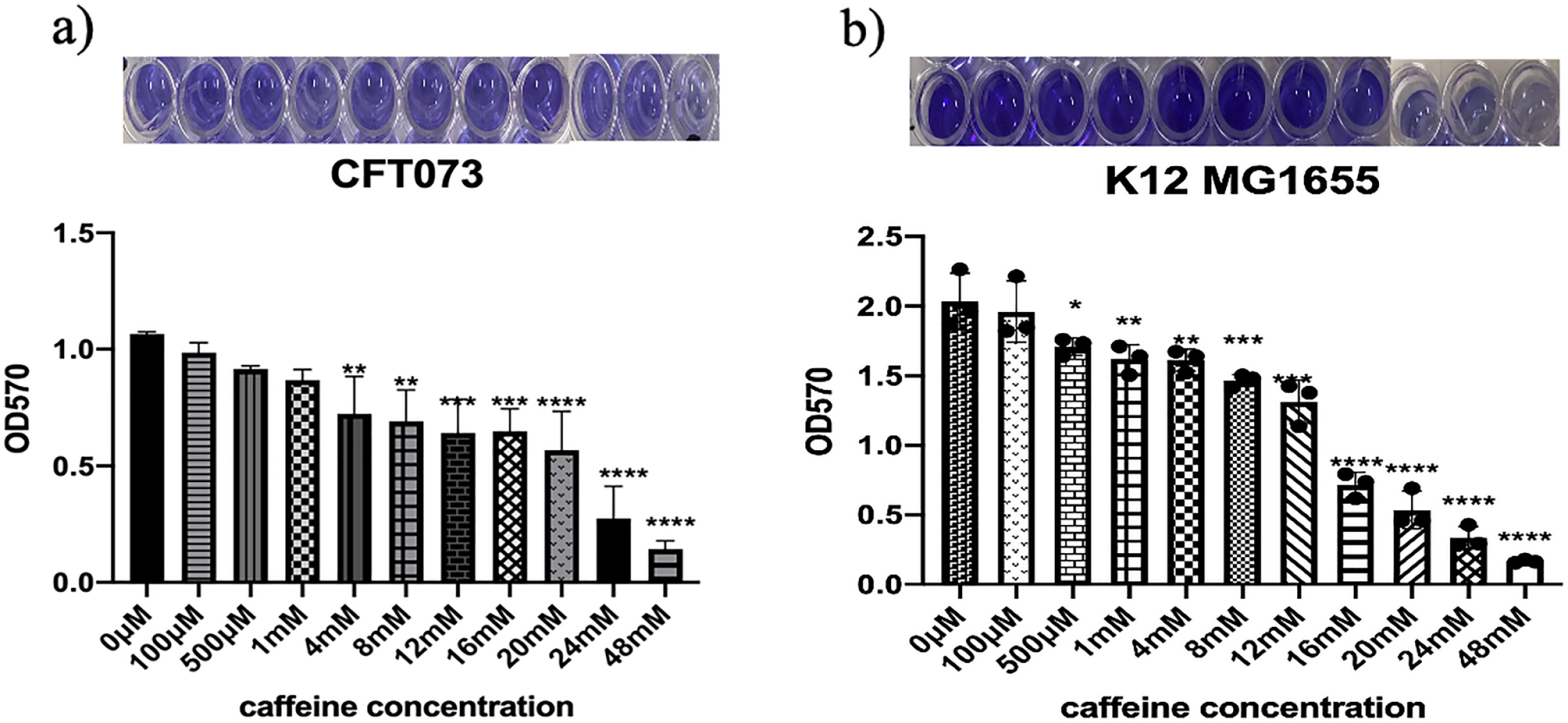
Effects of caffeine on CFT073 and K12 MG1655 biofilm formation. (**a**) Effect of caffeine on CFT073 biofilm formation at different concentrations determined by CV assay. Biofilm was allowed to form in the presence of caffeine for 48 h and results are expressed in terms of biofilm biomass, which was calculated by measuring optical density at 570 nm. (**b**) Effect of caffeine on K12 MG1655 biofilm formation at different concentrations determined by CV assay. The error bar represents the mean ± SD of the samples performed in triplicates where *p < 0.05, **p < 0.01, ***p < 0.001, and ****p < 0.0001 indicates statistically significant difference relative to the untreated control (one-way ANOVA).

CV assay is the most widely used method for biofilm quantification, which stains biofilm biomass^33,34^. The antibiofilm capability of caffeine was investigated by crystal violet (CV) assay with few modifications. The two strains, however showed different effects of caffeine on their biofilm-forming capability. The biofilm biomass was significantly higher in K12 MG1655 biofilm compared to CFT073. In CFT073, there was a constant decrease in biofilm formation as caffeine concentration increased from 100 μM to 4 mM. In the presence of 20 mM caffeine, biofilm formation was reduced to 53.29% (p < 0.0001). The reductions were more profound at 24 mM (27.58%) and 48 mM (13.41%) caffeine as compared to control(Fig. 2a). The inhibitory effect of caffeine is more significant on strain K12 MG1655 as the biofilm biomass was decreased to 8.19%at 48 mM caffeine as compared to the untreated control (Fig. 2b). This data indicates potent anti-biofilm capability of caffeine.

### Caffeine significantly reduced the survival and cytotoxicity of Uropathogenic *E. coli*

**Figure 3:**
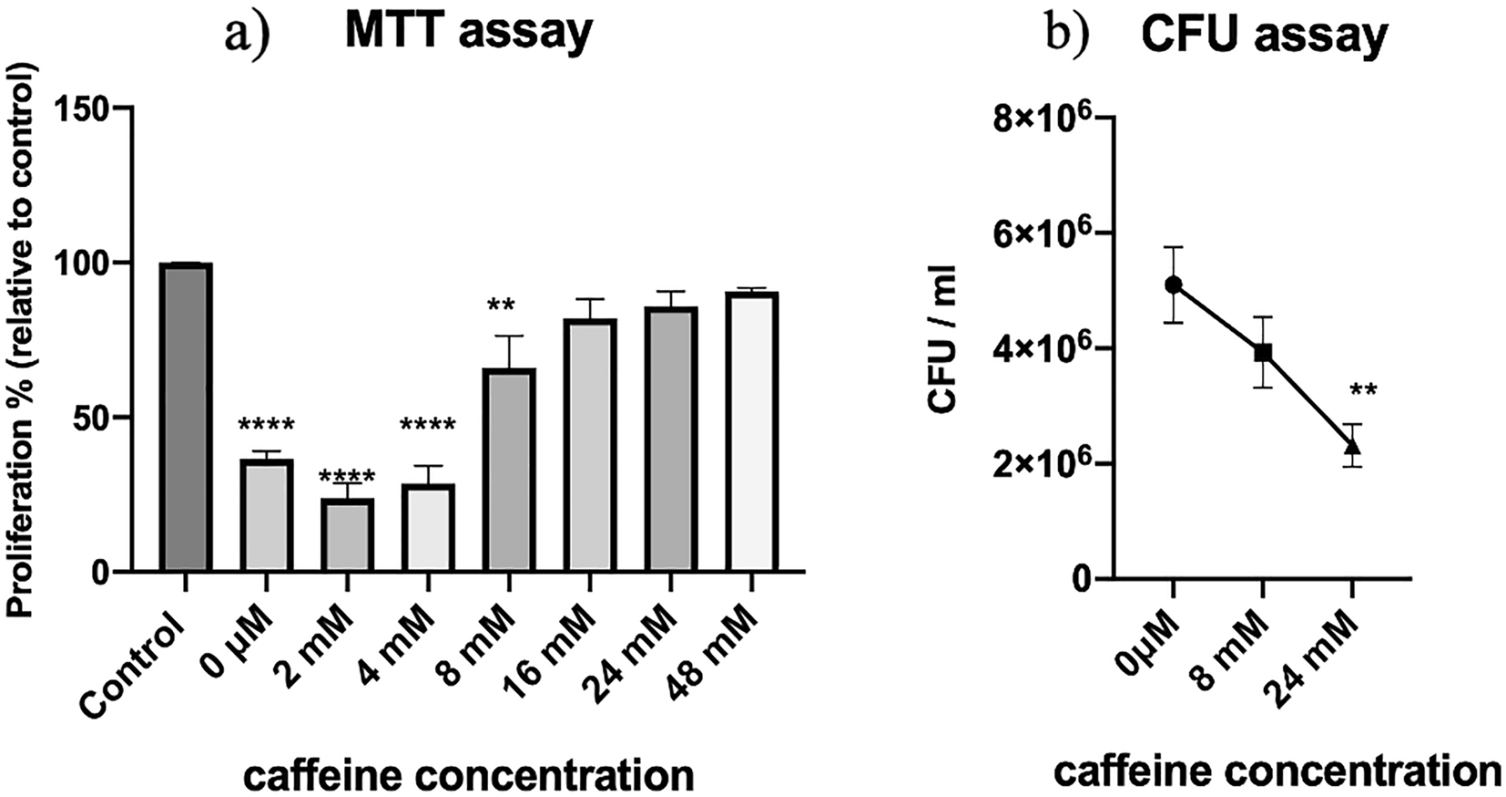
Effect of caffeine on uropathogenic *E.coli* using *in-vitro* co-culture model of T-24 cells CFT073. (**a**) Effect of untreated UPEC and caffeine treated UPEC on the viability of T-24 cells as determined by MTT assay. Only cells (T-24) served as control. (**b**) Effect of caffeine on the survival of UPEC inside T-24 cells. Untreated UPEC served as control. The experiment was performed in triplicates. The data represents an average of three independent experiments presented as mean ± SEM where * p < 0.05, **p < 0.01, ***p < 0.001, and ****p < 0.0001 indicates statistically significant difference relative to the untreated control (one-way ANOVA).

We further explored potential of caffeine against intracellular Uropathogenic *E. coli,* CFT073 using a gentamicin protection assay^35^. The UPEC were treated with different concentration of caffeine and then used for infecting T-24 cells. The cell proliferation of T-24 cells incubated with caffeine treated and untreated UPEC was evaluated by MTT assay^36^. The T-24 cell viability was reduced to 36.58 ± 4.361% when the cells were exposed to untreated UPEC CFT 073 (Fig. 3a). However, when the T-24 cells were infected with UPEC treated with 24 mM and 48 mM caffeine, the cell viability was increased to 85.90 ± 8.255% and 90.67 ± 1.965% respectively as compared to untreated UPEC (Fig. 3a). We further evaluated the intracellular bacterial content of T-24 cells incubated with UPEC exposed to 8 mM and 24 mM caffeine. The bacterial count was reduced to approx. 2 × 10^6^ cfu/ml when cells infected with UPEC treated with 24 mM caffeine as compared to 5 × 10^6^ cfu/ml in untreated UPEC CFT 073. The results indicated that caffeine significantly reduced the survival of UPEC at 8 mM and 24 mM (Fig. 3b). These results demonstrated that the caffeine attenuated the cytotoxic effects of UPEC on bladder epithelial cells. In addition, caffeine reduced the survival potential of Uropathogenic *E. coli*.

### Fluorescence microscopy confirmed the inhibition of biofilm forming capability of *E. coli* by caffeine

**Figure 4:**
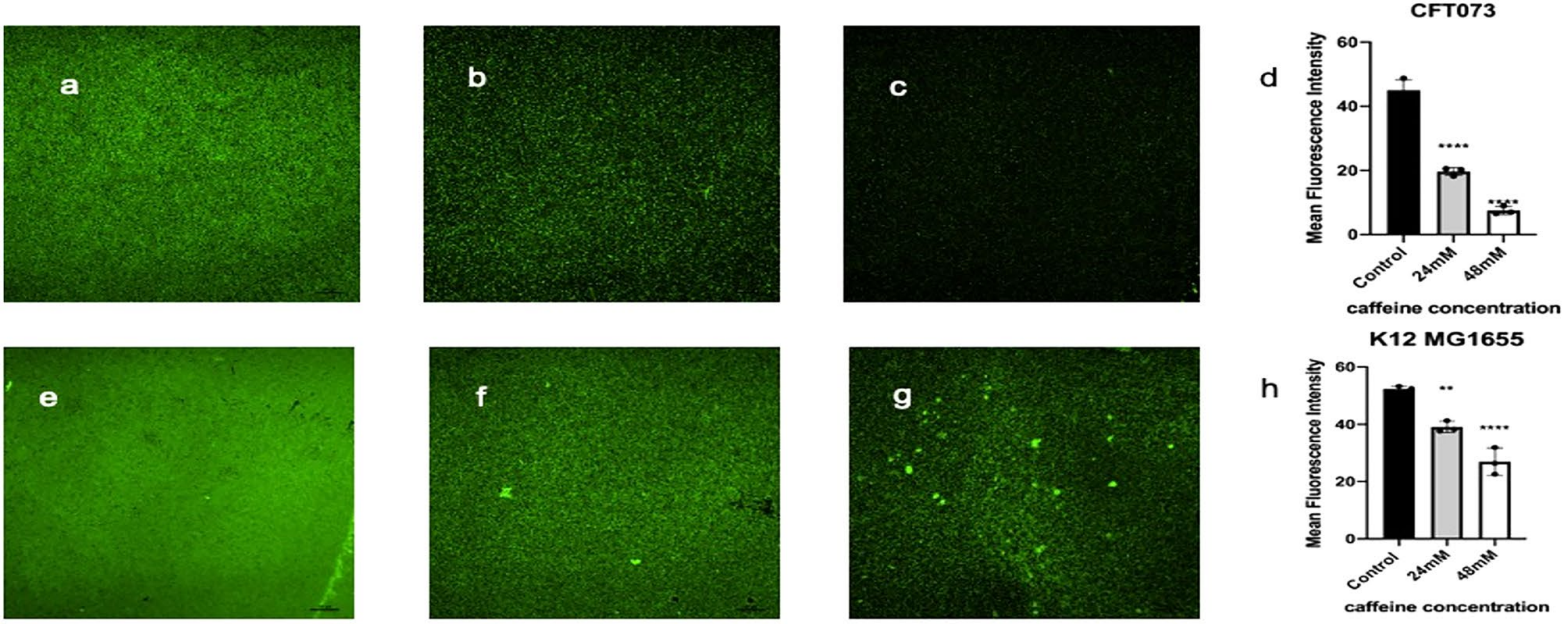
Effects of caffeine on CFT073 andK12 MG1655 biofilms as determined by fluorescence microscopy (FM). Syto9 stained live bacteria and emit green fluorescence intensity. FM images ofCFT073 biofilms, (**a**) without caffeine; (**b**) with 24 mM caffeine; (**c**) with 48 mM caffeine; (**d**) quantitative analysis of fluorescence image ofCFT073 biofilm using ImageJ and GraphPad prism8. FM images of K12 MG1655 biofilms (**e**) without caffeine; (**f**) with 24 mM caffeine; (**g**) with 48 mM caffeine; (**h**) quantitative analysis of fluorescence image of K12 MG1655 biofilm using ImageJ and GraphPad prism8.

The effect of caffeine on CFT073 and K12 MG1655 biofilms was further observed by fluorescence microscopy. The fluorescence images of SYTO 9 staining indicated that caffeine effectively reduced the biofilm biomass of both strains. The mean fluorescence intensity was decreased considerably even at lower concentrations of caffeine(8 mM) and decreased to 16.68%at 48 mM caffeine as compared to control(p < 0.0001) in CFT073 biofilm. (Fig. 4a–d). The mean fluorescence intensity of K12 MG1655 biofilm was not significantly reduced at lower concentrations of caffeine till 8 mM but reduced to appx. 50% at 48 mM caffeine as compared to control(p < 0.0001) (Fig. 4e–h). The microscopic observations confirmed the caffeine’s ability to inhibit *E. coli* biofilms.

### SEM analysis demonstrated that caffeine efficiently impeded CFT073 and K12 MG1655 biofilms

**Figure 5:**
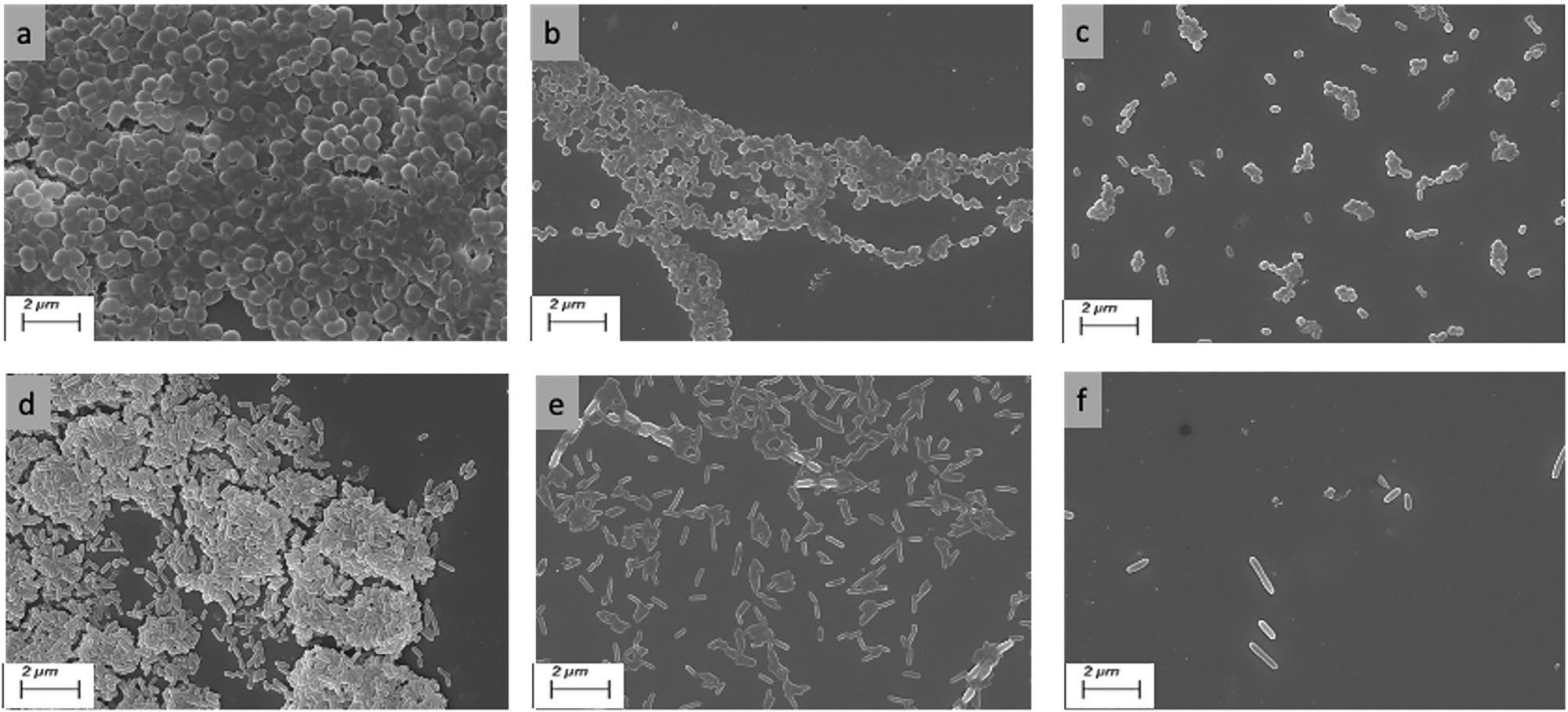
Dose dependant effects of caffeine on *E. coli* biofilms observed under SEM.SEM images of CFT073 biofilms on glass slips, (**a**) without caffeine; (**b**) with 24 mM caffeine; (**c**) with 48 mM caffeine; SEM images of K12 MG1655 biofilms on glass slips (**d**) without caffeine; (**e**) with 24 mM caffeine; (**f**) with 48 mM caffeine. Magnifications and bar markers are ×10,000 (**a**) ×5000 (**b–f**) and the scale is 2 μm long.

The effect of caffeine on *E. coli* biofilms was further confirmed by SEM analysis. The biofilm of both bacteria was formed on a glass coverslip with different concentrations of caffeine and analyzed under SEM. The CFT073 bacteria without caffeine form well-defined biofilm and are entrapped in an extracellular matrix (Fig. 5a). There was a significant reduction in the bacterial biofilm-forming ability of CFT073after 24 mM caffeine treatment (Fig. 5b), and only a few bacteria were visible after 48 mM caffeine treatment (Fig. 5c). A similar trend was observed in K12 MG1655, with more bacteria forming robust biofilms with a well-defined matrix of polymeric substances in the control set compared to fewer bacteria at 24 mM and 48 mM caffeine (Fig. 5d–f). The SEM images clearly depicted the reduced biofilm formation of *E. coli* in the presence of caffeine.

### Caffeine strongly binds with curli regulator protein (csg D)

**Figure 6:**
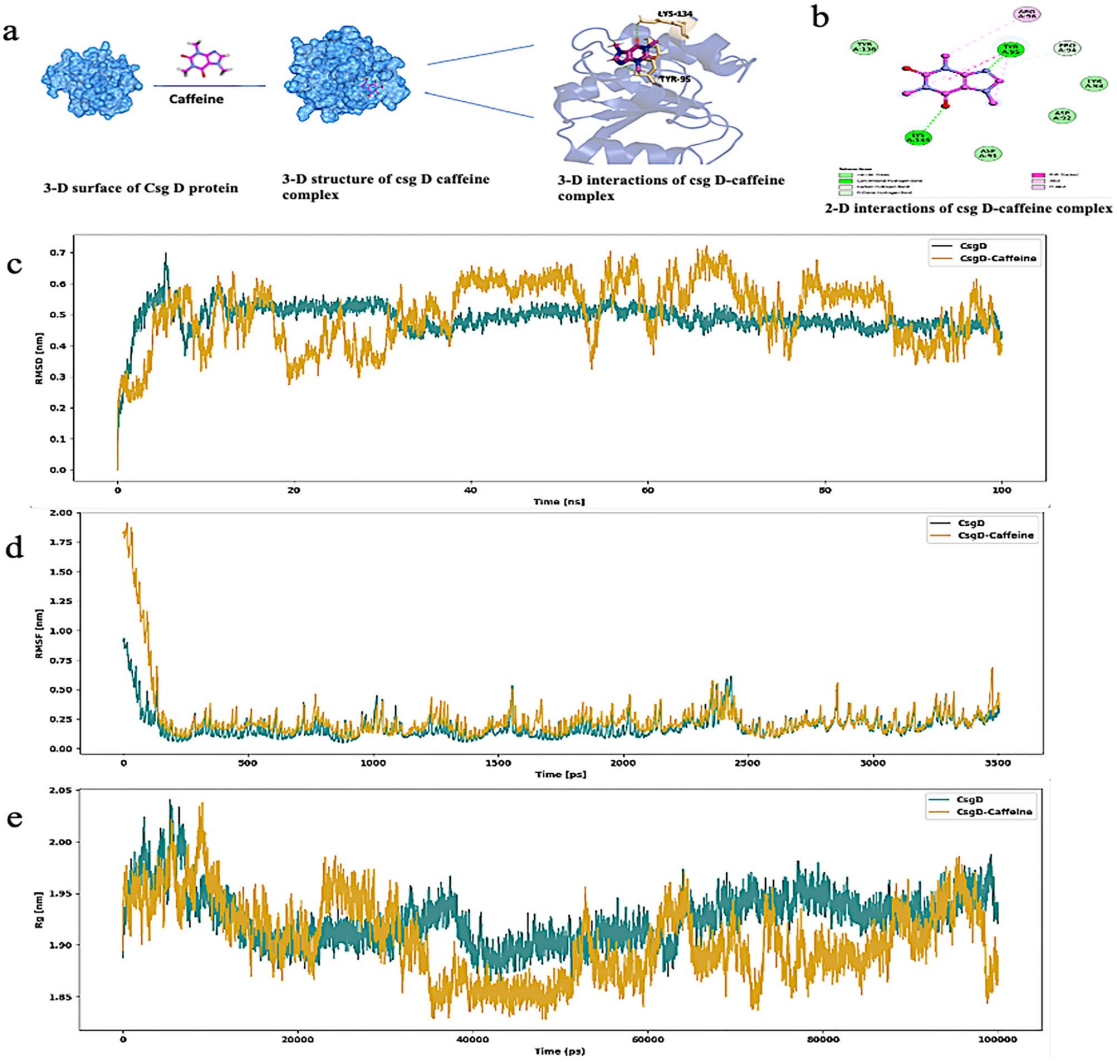
(**a**) Three-dimensional visualisation of caffeine interaction onto the active site of curli regulator protein (csg D). Figure represents 3-D structure of csg D, caffeine, docked complexes (**b**) 2-D protein–ligand hydrogen bond interactions. Result from 100 ns molecular dynamics simulation of docked complex. (**c**) RMSD fluctuation**,** (**d**) RMSF value**,** (**e**) radius gyration.

Curli fibers play a major role in the structure of biofilm as they are the adhesive filaments that promote attachment to both biotic and abiotic surfaces. Additionally, they form the basis of Gram-negative bacteria's biofilm extracellular matrix^12,37^. Curli is encoded by csgBA and csgDEFG operon and csg D is the master regulator of curli biogenesis. It initiates the transcription of other curli-specific genes (csg A and csg B) and is responsible specifically for its sessile transition during biofilm formation^16^. Since caffeine showed substantial antibiofilm property as shown in previous results, we further studied the interaction of caffeine with curli regulator protein (csg D). We did a molecular docking analysis of caffeine with csgD protein. The study analysed caffeine ligand for their binding affinity and intermolecular interactions against the CsgD protein. The binding affinities of the caffeine ligand with CsgD protein was found to be − 7.11 kcal/mol reflecting the stability of interactions, whereas their total energy was found to be 32.20 kcal/mol. The caffeine ligand molecule showed hydrogen bonding (H bond) with the CsgD residues, Lys134 and Tyr95 (Fig. 6a). The other interactions of caffeine with CsgD like pi–alkyl, pi–pi and Vander Waals interactions are shown in Fig. 6b.

Molecular dynamics simulation is a computational method for studying protein and ligand complex structural stability and molecular behaviour. GROMACS software was used to perform MD simulations of unbound CsgD protein and docked CsgD-ligand complexes at 100 ns. Root mean square deviations (RMSD), root mean square fluctuations (RMSF), solvent-accessible surface area (SASA), and radius gyration (Rg) were measured in high-binding-affinity complexes of caffeine ligands and CsgD proteins.

The binding energy and number of hydrogen bonds formed between the CsgD protein and caffeine ligands were used to determine the interaction. According to the MD simulation results, the unbound CsgD had lower RMSD fluctuations than the complexes. The RMSD of the ligand-bound CsgD complex fluctuated until 90 ns before plateauing (Fig. 6c). After 90 ns, the RMSD values of the ligand complex are more stable due to their ability to form stable complexes with ligands, showing high potential as inhibitors. The RMSD of caffeine was erratic until 90 ns,and thereafter it got stabilised and remained stable up to 100 ns simulations.

The RMSF values represent each residue's thermodynamic stability and degree of movement. The apo form of CsgD did not show any significant fluctuations, which represents the absence of a ligand. However, when the ligand is bound to CsgD protein, the flexible residues in the ligand-binding regions were shown to shift to accommodate the ligands and maintain equilibrium (Fig. 6d).

The Rg analysis reveals the stability of each molecule and the structure's overall dimension and compactness. Initial, the Rg value of CsgD with caffeine ligand was relatively low. Later, both the apo and holo forms of protein were stabilised. However, CsgD protein and CsgD-caffeine complex remained stable almost throughout the simulation. The ligand-complex Rg-value varied from 1.85 to 2.04 nm (Fig. 6e) and was extremely close to the Rg-value of apo CsgD. After 35 ns, the protein complex achieved a more stable conformation than the unbound form, which shows the ligand binding contribution toward the stability of the complex.The in-silico docking analysis aligning with our wet lab results and indicated that caffeine has a high potential to inhibit biofilm formation through regulation of csg D, the crucial activator required for the expression of curli subunits.

### Caffeine inhibited the expression of curli specific genes in both the strains of *E. coli*, CFT073 and K12 MG1655

**Figure 7:**
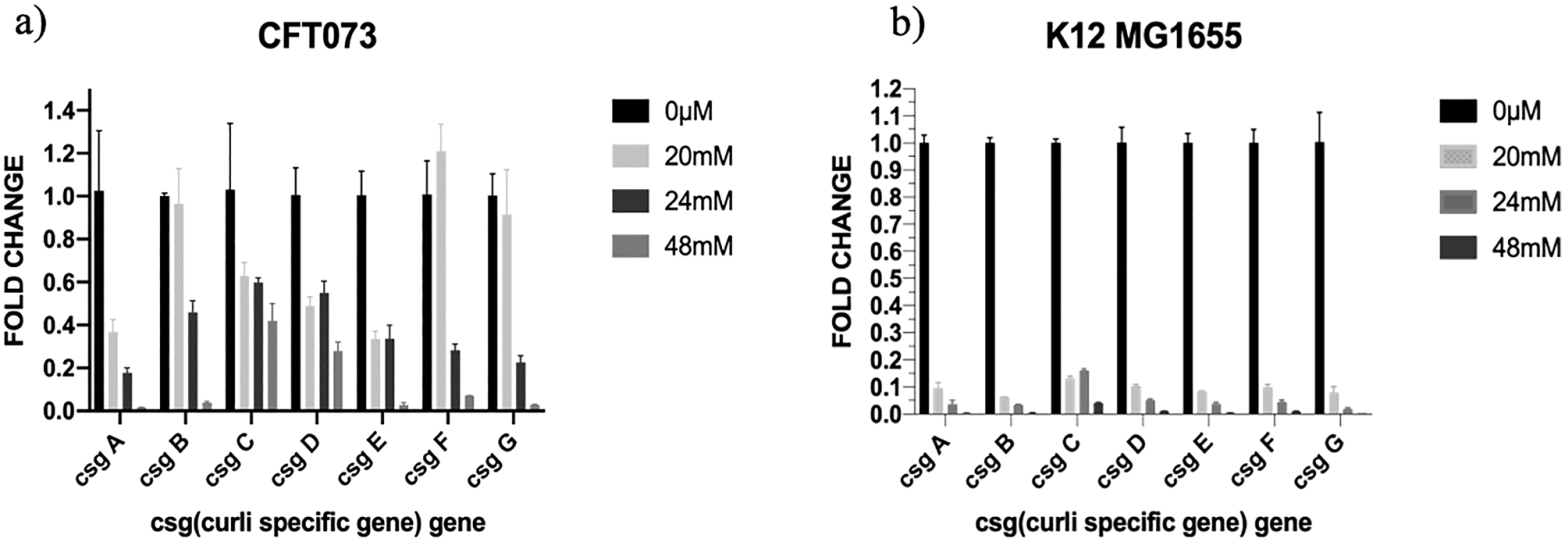
Effect of caffeine on *E. coli* curli genes expression (**a**) qRT-PCR results of curli-specific genes with caffeine (20 mM, 24 mM, 48 mM) treatment and without caffeine in *E. coli* CFT073 biofilms. (**b**) qRT-PCR results of curli-specific genes with caffeine (20 mM, 24 mM, 48 mM) treatment and without caffeine in *E. coli* K12 MG1655 biofilms. Error bar represents the mean ± SD of the samples performed in triplicates where **** indicates a statistically significant difference (p < 0.0001) relative to the untreated control (two-way ANOVA control; 0 μM).

We further evaluated the transcription of curli-specific genes, which code for a curli protein responsible for the adhesion and maturation of biofilms in the presence of caffeine. This was performed to prove the hypothesis that caffeine can inhibit curli formation by decreasing curli-specific gene expression. The *E. coli* biofilms were grown in a six-well plate at 28 °C for 48 h, supplemented with different concentrations of caffeine. qRT-PCR data revealed that the expression of all the curli-specific genes decreased significantly in CFT073 biofilms. The expression of structural components of curli genes (csg A and csg B) were drastically reduced in the presence of caffeine (Fig. 7a). However, the expression of csg C decreased to 0.4188 ± 0.082 at 48 mM caffeine compared to 1.030 ± 0.308 in control. In K12 MG1655 biofilms, the expression of all the curli-specific genes was significantly decreased at 20 mM, 24 mM, and 48 mM caffeine compared to control (p < 0.0001). Furthermore, the expression of curli-specific genes exhibited a dose-dependent decrease compared to control (Fig. 7b). The qRT-PCR results confirmed that caffeine strongly decreased the transcription of curli genes and, consequently, the formation of biofilms in CFT073 and K12 MG1655.

## Discussion

Bacterial biofilms can have adverse impacts on human health and researchers in recent years have focused on preventing and treating biofilm-related infections^38^. Biofilms can be compared to “cities of microbes”, and EPS is their "house", with the most significant conserved components of *E. coli* matrix being proteinaceous curli fibers, flagella, and cellulose^39–41^. It is also important to note that antibiotic resistance is a significant problem associated with biofilm infections, which urges us to find alternative treatments. Researchers have increasingly turned to natural products and rediscovering traditional compounds as a solution to chronic biofilm-related infections owing to increasing antibiotic resistance^40–42^. In this light, we studied the efficacy of caffeine at inhibiting *E. coli* biofilm formation and the potential mechanism behind the anti-biofilm property of caffeine.

Previous studies have shown that caffeine inhibited the growth of *Escherichia coli*, *Enterobacter aerogenes, Proteus vulgaris, and Pseudomonas aeruginosa*^23,43^. Our results concord with Al-Janabi^31^, who stated that caffeine at 10 mg/ml could inhibit most of the bacteria (especially *S aureus*, *E cloacae*, and *E aerogenes*) than ampicillin. The present study also showed that caffeine eliminated over 90% of both pathogenic (UPEC) and commensal (*E. coli* K12 MG1655) bacteria in the planktonic form at 48 mM caffeine (Supplementary Fig. S1a,b). However, the MIC variation was attributed to the different strains taken in the study (Supplementary Fig. S1a,b). Moreover, the MBIC of caffeine is 1.6 times the MIC of UPEC and 1.25 times the MIC of *E. coli *K12 MG1655 (Supplementary Fig. S1). This might be due to the presence of a biofilm matrix that will hinder the antimicrobial agent from acting. The growth rate of *E. coli* was examined in the presence of caffeine, and the results illustrated that caffeine inhibited the growth of *E. coli* in a dose-dependent manner (Fig. 1d,e). We observed that the lower concentrations of caffeine (100 μM to 1 mM) were insufficient to alter the cell viability, but higher concentrations (20 mM, 24 mM, and 48 mM) of caffeine significantly reduced the viability of both bacteria. (Fig. 1b,c). Together, these observations showed that caffeine could inhibit *E. coli* biofilm by preventing bacterial proliferation, thus eliminating it.

It is also important to eliminate pathogens within urinary epithelial cells to prevent recurrent UTIs as intracellular bacteria mostly contribute to UTIs^44^. In order to determine the effect of caffeine on the survival and cytotoxicity of UPEC in the presence of caffeine, we measured the intracellular bacterial count of Uropathogenic *E. coli* CFT073 using T 24 cell line as an in vitro infection model, as well as evaluated its potential cytotoxicity. According to our findings, caffeine reduced the survival of UPEC CFT073 within human bladder epithelial cells (Fig. 3b). Also the cell viability of T-24 cells was not affected in the presence of caffeine-treated UPEC (Fig. 3a). In uropathogens, antibiotic resistance increases with the maturation of biofilms. There is a high prevalence of biofilms in the urinary tract, where uropathogens persist and cause recurrent urinary tract infections^45^. The present study led us to speculate that caffeine might be an option as a treatment measure in UTIs where biofilms are involved.

The CV results demonstrated that caffeine annihilated the biofilm-forming ability of CFT073 bacteria to a great extent, as the biofilm biomass was reduced by 86.58% at 48 mM caffeine (Fig. 2a). A striking observation was that K12 MG1655 biofilms had almost twice the biomass of UPEC biofilms, and caffeine still dramatically reduced K12MG1655 biofilms by almost 90% at 48 mM (Fig. 2b). We further confirmed these results by fluorescence microscopy. The biofilm staining was done with SYTO 9 and the mean fluorescence intensity dropped considerably to (7.5 ± 1.2) at 48 mM caffeine compared to (45.05 ± 3.2) in the CFT073 biofilm (Fig. 4a–d). The fluorescence microscopy images revealed that caffeine significantly suppressed biofilm formation of K12 MG1655 bacteria (Fig. 4e–h). We next verified the effect of caffeine on biofilms by SEM analysis. The SEM results were in accordance with previously discussed quantitative and qualitative results as we see that caffeine significantly abated the biofilm-forming ability of both bacteria at 48 mM caffeine suggesting a reduction in matrix formation (Fig. 5). All these data suggested that caffeine has antibiofilm activity against UPEC and *E. coli* K12 MG1655.

**Figure 8:**
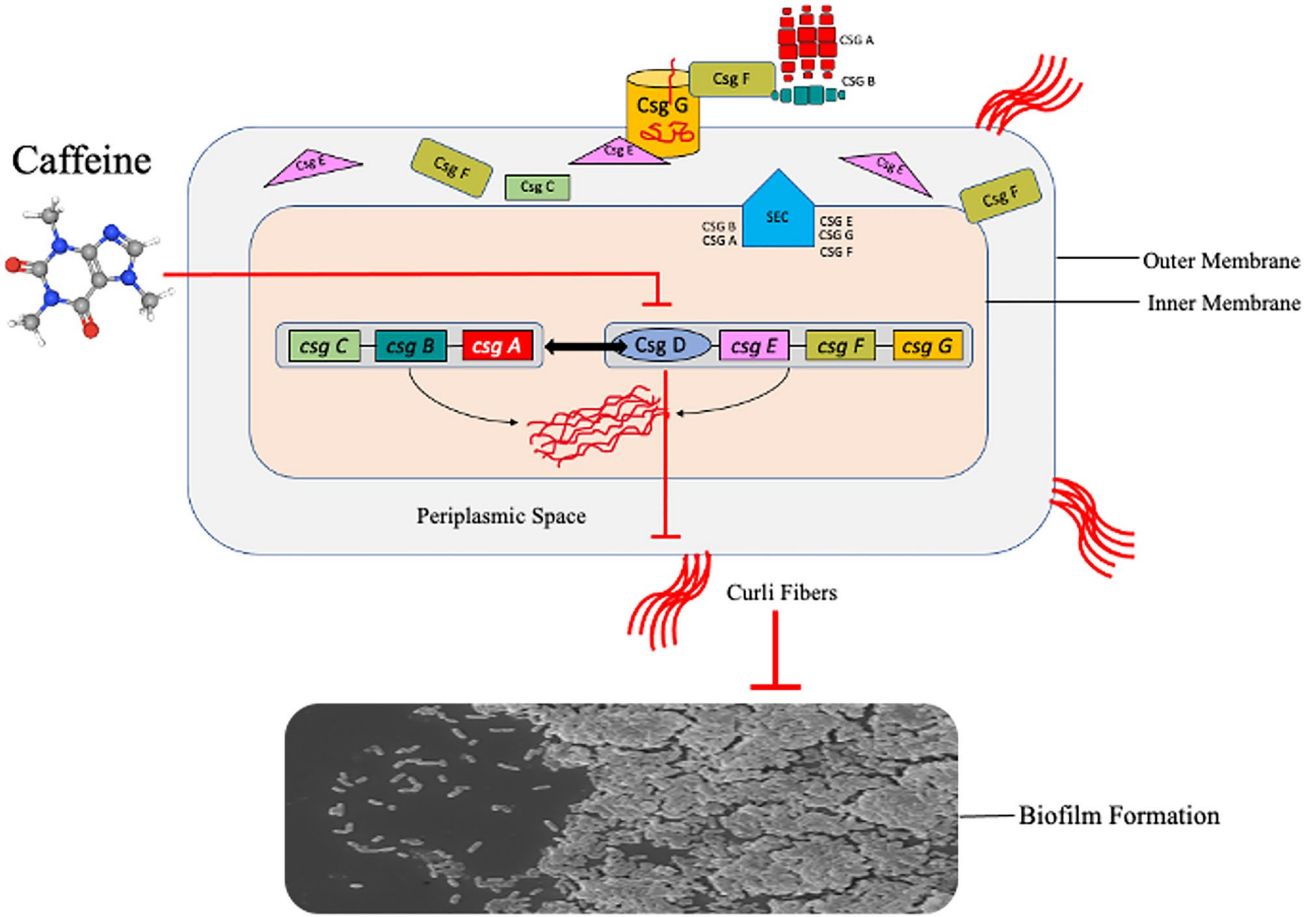
Schematic representation of inhibition of *E. coli* biofilm by Caffeine. Csg D promotes curli biogenesis, leading to the production of curli fibers and the formation of robust biofilms. Csg A and Csg B are major structural units, while Csg E and Csg F are accessory proteins that promote curli subunit transport through Csg G pores. In the periplasm, CsgC prevents CsgA from polymerizing. A sec signal sequence is encoded for translocation into the periplasm by all the proteins encoded by the csg operons, except for CsgD. In the diagram above, red arrows indicated the result of adding caffeine and demonstrated that it decreased the production of curli and *E. coli* biofilms.

Biofilm formation is mediated by a various structural virulence factor such as type 1 fimbriae, curli, pili and flagella^46–48^. The curli plays a vital role in the progression of UTIs caused by *E. coli*. It has been shown that curliated bacteria adhere better to the uroepithelial cells than bacteria without curli, as well as their ability to persist in urinary tracts, thus contributing to UTIs^10,12,13,49,50^. When *Escherichia coli* shifts from being planktonic to the sessile mode in biofilm, the flagellum formation stops, thereby boosting the production of curli fimbriae and polysaccharides to enhance adhesion between cells^9,13,51^. It intrigued us to determine the potential molecules against the master regulator of curli biogenesis, Csg D. We performed docking analysis of caffeine with the active sites of Csg D, which showed that caffeine strongly binds to Csg D with the binding affinity of −7.11 kcal/mol. We further studied the structural stability of caffeine -csg D complex by MD simulation studies which determined the stability of the complex up to 100 ns simulations. Results showed that the anti-biofilm activity of caffeine might be related to curli expression via curli regulator protein, csg D (Figs. 6, 8).Recently, Serra et al. reported that epigallocatechin gallate (EGCG) reduced the *E. coli* biofilm matrix by blocking curli assembly via interfering with the expression of csg D^52^. Furthermore, we examined whether caffeine could influence the expression of curli-related genes in the csg family by qRT-PCR. The mRNA expression levels of csg A, csg B, csg C, csg D, csg E, csg F and csg G were downregulated significantly after caffeine treatment at 24 mM and 48 mM in both strains (Figs. 7a,b, 8). So, we may speculate that caffeine inhibited curli production at the transcriptional level as both csg A and csg B (structural components of curli) mRNA expression level was downregulated significantly in both biofilms (Fig. 8).

Previously, it has been reported that many natural compounds like EGCG^52,53^, eugenol^54^, cinnamaldehyde^55^, ginkgolic acids^56^, coumarins^57,58^ inhibit biofilm formation by impeding curli fimbriae production. The presented study is an extension of the effort to find natural compounds which can demolish these complex biofilm structures. It can be inferred in the framework of this study that the antibiofilm activity of caffeine is curli mediated in *E. coli*. It has been documented that an Espresso coffee contains about 240–720 mg caffeine in 8 oz, which corresponds to 5 mM to 15 mM^59^. Interestingly, the present study demonstrated the significant antibiofilm effect against UPEC starts from 4 mM caffeine (Fig. 2a). A moderate daily caffeine intake of 400 mg in an average adult is recommended without any adverse effects^60,61^. The use of caffeine as an adjuvant therapy for UTI patients may prove effective based on such observations. The effects of caffeine, however, vary according to body weight and caffeine sensitivity. Taking our results into consideration, caffeine can be a potential antibiofilm agent against *E. coli* biofilms and may provide an alternative therapeutic strategy for treating *E. coli* biofilm-related infections.

## Methods

### Materials, bacterial strain and culture conditions

Caffeine was purchased from Sigma-Aldrich (cat # C0750, Saint Louis, MO, United States). The bacterial strains used were CFT073, UPEC strain (ATCC # 700928; United States) and K12 MG1655 (ATCC # 700926; United States) which were first streaked onto fresh plates from glycerol stocks stored in −80 °C. Single colony was then picked and grown in Luria–Bertani (LB) (SRL Cat# 29817, India) media at 37 °C for 24 h with continuous shaking at 150 rpm to obtain primary culture. For all the experiments 10^7^ CFU/ml *E.coli* suspensions were used to ensure similar number of bacteria in each experiment. Other chemicals used were 3-(4,5-Dimethylthiazol-2-yl)-2,5-Diphenyltetrazolium Bromide-MTT(Invitrogen Cat # sM6494; United States), Trypsin–EDTA solution (1×) (Himedia Cat # TCL048)for cell viability of bacteria. For RT PCR, TRI Reagent (Sigma Cat # T9424; United States) was used, Revert Aid First Strand cDNA Synthesis Kit (Thermo Scientific Cat # K1622; United States) and SYBR Green Master Mix (Applied biosystems Cat# A25742; United States) were used. Crystal violet (SRL Cat# 28376, India) was used for CV assay and SYTO9 green fluorescent nucleic acid stain (Invitrogen Cat # S34854, United States) for fluorescence microscopy.

### Cell line and culture conditions

The human urinary bladder epithelial cell line T-24 (Cat # HTB-4, ATCC, Manassas, VA, United States) was used for co-culture experiments. The T-24 cells were grown in McCoy’s 5A medium supplemented with 10% heat inactivated FBS (Gibco Cat #10270106, Brazil) and incubated at 37 °C with 5% CO_2_ in humified incubator.

### Determination of minimum inhibitory concentration (MIC) and minimum biofilm inhibitory concentration (MBIC)

Minimum inhibitory concentration (MIC) was determined by the broth dilution method with a few modifications described by Sivaranjani et al.^62^. Caffeine was added to 1 × 10^7^ cfu/ml *E. coli* suspensions in LB media at various concentrations (100 μM, 500 μM, 1 mM, 4 mM, 8 mM, 12 mM, 16 mM, 20 mM, 24 mM and 48 mM) in 15 ml falcon tubes. Bacterial suspension without caffeine served as a control set. The tubes were incubated at 37 °C for 24 h, and the optical density was measured at 600 nm using a spectrophotometer (TECAN Infinity 200 pro). MIC is the minimum concentration of caffeine required to inhibit the growth of planktonic *E. coli* cells after 24 h incubation. For determination of MBIC, caffeine was added to 10^7^ cfu/ml *E. coli* suspensions at the different concentrations (100 μM, 500 μM, 1 mM, 4 mM, 8 mM, 12 mM, 16 mM, 20 mM, 24 mM and 48 mM) in 12-well tissue culture plate. As a control set, bacterial suspensions without caffeine were used. The plates were incubated at 37 °C for 48 h, and the optical density was measured at 600 nm using a spectrophotometer. MBIC is the minimum caffeine concentration required to inhibit biofilm cell growth after 48 h incubation.

### Growth kinetics

*E. coli* CFT073 and *E. coli* K12 MG1655 cultures were inoculated with new colonies and incubated overnight at 37 °C on orbital shaker. 1 × 10^7^ CFU/mL of overnight cultures were incubated with different concentrations of caffeine (100 μM, 500 μM, 1 mM, 4 mM, 8 mM, 16 mM, 20 mM, 24 mM and 48 mM) in a 96-well plate (SPL Lifesciences, cat #32096) at 37 °C for 4 h. Growth kinetics curves were obtained by measuring the optical density at 600 nm at each time point from 15 min to 4 h using a microplate reader (TECAN Infinity 200 pro) with continuous linear shaking.

### Determination of cell viability of bacterial cells in biofilm

The cell viability was checked by MTT assay^33^. E*. coli* CFT073 and *E. coli* K12 MG1655were grown overnight to obtain primary culture. The experiment was conducted further using overnight cultures in the log phase at OD600 between 0.6 and 0.8. The culture (1 × 10^7^ CFU/mL) was then incubated with different concentrations of caffeine (100 μM, 500 μM, 1 mM, 4 mM, 8 mM, 12 mM, 16 mM, 20 mM, 24 mM and 48 mM) in a 96-well plate(SPL Lifesciences, cat #32096) at 37 °C for 24 h. After that, 20 μl of MTT (5 mg/ml) was added to each well in 200 μl LB media and incubated at 37 °C for 2 h. After discarding the media, 100 μl DMSO was added and incubated at 37 °C for 20 min. The Optical density was measured at 540 nm using a microplate reader (TECAN Infinity 200 pro).

### Determination of anti-biofilm activity by crystal violet assay

Christensen first described CV assay and has improved constantly to be suitable for all biofilm quantification^63,64^. The antibiofilm activity was checked by crystal violet assay as described. *E. coli* CFT073 and *E. coli* K12 MG1655 cultures were inoculated with fresh colonies and incubated overnight at 37 °C in LB media. 1 × 10^7^ CFU/mL of overnight cultures were incubated with different concentrations of caffeine (100 μM, 500 μM, 1 mM, 4 mM, 8 mM, 12 mM, 16 mM, 20 mM, 24 mM and 48 mM) in a 96-well plate (SPL Lifesciences, cat #32096) at 28 °C for 48 h. The culture was washed with 1× PBS three times for quantification to remove planktonic bacteria. The biofilm formed was then heat-fixed at 60 °C for 1 h. The formed biofilms were then stained with 0.1% crystal violet for 30 min. After that, it was washed with autoclaved water and solubilized in 95% ethanol and further the Optical density was measured at 570 nm using a microplate reader (Tecan Infinity 200 pro).

### Determination of cell viability using co-culture of T-24 cell line with uropathogenic *E. coli* CFT073

The effect of caffeine treated UPEC on T-24 cell viability was assessed in co-culture model by MTT assay. Briefly T-24 cells (2 × 10^4^ cells per well in 200 μl McCoy’s media) were seeded in 96 well plate at 37 °C in a humidified CO_2_ incubator. The CFT073 biofilm was formed with different concentration of caffeine (0 μM, 2 mM, 4 mM, 8 mM, 16 mM, 24 mM and 48 mM) in a 96-well plate (SPL Lifesciences, cat #32096) as described in above section. The T-24 cells were infected in triplicates with and without caffeine treated CFT073 biofilm bacteria for 24 h at 1:5 MOI. After incubation, the media was discarded and 20 μl MTT (5 mg/ml) was added in 200 μl McCoy’s media and incubated for 2 h at 37 °C in a humidified CO_2_ incubator. The media was then discarded and the formed formazan crystals were dissolved in 100 μl DMSO. The Optical density was measured at 540 nm using a microplate reader (Tecan Infinity 200 pro). The experiment was repeated three times.

### Determination of intracellular colony forming units of uropathogenic *E. coli* in co-cultures with T-24 cells—infection assay

The effect of caffeine was determined on the virulence of uropathogenic *E. coli* by measuring the intracellular CFU count on co-culture with T-24 cells. The human urinary bladder epithelial cell line T-24 (1 × 10^6^ cells per well) were seeded in six-well plate. The cells were then allowed to form a confluent monolayer in the plates and then infected with CFT073 biofilm bacteria (MOI 1:5; 1 OD—8 × 10^8^ bacteria) treated with and without caffeine for 1 h. The cells were then treated with media having antibiotic (gentamycin, 200 µg/ml) for 1 h to eliminate extracellular bacteria and thereafter incubated for an additional 2 h in media containing antibiotic (gentamycin, 20 µg/ml). After 2 h, the cells were washed with 1× PBS and lysed in 0.025% SDS. The cell suspensions were diluted 10^1^–10^5^ times and plated on LB agar plates followed by incubation at 37 °C for 12–16 h to calculate the relative CFU/ml.

### Fluorescence microscopy

Bacterial biofilm was formed as described in the CV assay. The planktonic bacteria were removed, and the formed biofilm was stained with 1.5 μM SYTO 9 green-fluorescent nucleic acid stain and incubated in darkness for 15 min. The plate was then washed with 1× PBS and visualized under a fluorescence microscope (Motorized Inverted Microscope. Ii2; Nikon). Quantitative analysis was then performed with Image J software.

### SEM analysis

Biofilm was formed with respective bacteria with different concentrations of caffeine in six-well microtiter plates (Genetix, cat #30006), each containing a round glass coverslip of 12 mm at 28 °C for 48 h. The control set had bacteria without caffeine. After incubation, washing was performed with 1× PBS, and the coverslips were fixed overnight with 2.5% glutaraldehyde at 4 °C, followed by dehydration in graded ethanol. The sample was then airdried, sputter-coated with gold, and examined under SEM.

### Docking analysis of curli regulator protein with caffeine

#### (a) CsgD protein and ligand structure preparation

The structure of the caffeine ligand (PubChem CID: 2519) was retrieved in SDF format from the PubChem database^65^. Using the Open Babel tool^66^, a two-dimensional (2D) SDF file of a caffeine ligand was transformed into a three-dimensional (3D) PDB file format. The three-dimensional structure of the biofilm regulator CsgD regulatory protein of *Salmonella enterica* (PDB ID: 5XP0) was downloaded from the RCSB protein structure database^67^. csg D structure was refined by adding polar hydrogen atoms and removing water molecules using the protein preparation wizard of Discovery Studio 2021^68^ to perform docking and molecular dynamics simulation studies.

#### (b) Molecular docking and molecular dynamics simulation studies

To identify the potential binding location, binding interactions, and affinity, molecular docking of the caffeine ligands was carried out against CsgD protein structure. Caffeine ligands and the protein structure of *Salmonella enterica's* CsgD were inputed into the Dock Thor tool for blind docking using the default settings^69^. Molecular dynamic simulations were performed using the CHARMM36 force field^70^in the GROMACS 2020.3^71^ package to understand better the behaviour of the apo CsgD and docked complexes (CsgD-Caffeine) in biological environments. The CGenFF server was used to generate the caffeine ligand topology files^72^. The periodic boundary condition (PBC) was set at 2 nm from each face, and the docked complex was positioned in the middle of a box made of a dodecahedron. Using a simple point charge (SPCE) water model, the area around the protein–ligand complexes was filled, and the system was neutralised by swapping out precisely equal amounts of water molecules with counterions (Na^+^). The system's energy minimisation was performed using the steepest descent algorithm until a tolerance of 1000 kJ/mol/nm was attained. Later, whole systems were simulated for 100 ns (ns). Xmgrace graphical tool was used to create simulation graphs.

### RNA isolation and q- RT PCR analysis

Bacterial biofilms were formed in six-well microtiter plates (Genetix, cat #30006) with different concentrations of caffeine at 28 °C for 48 h, and total RNA was extracted from the culture using TRI reagent. The quantification of RNA was done using multiscan sky microplate spectrophotometer and 1000 ng RNA was subjected to c-DNA synthesis using Revert Aid cDNA kit according to manufacturer’s instructions. The gene-specific primers were designed using a gene runner and further checked by PRIMER BLAST, and 16 s RNA served as the internal control(Supplementary Table S1)^33^. The gene expression of curli-specific genes with and without caffeine was checked using q RT-PCR (ABI real-time PCR system). The reaction was carried out with SYBR green (Applied Biosystems) in 96 well plate and a 15 μl reaction volume. The relative gene expression was calculated by 2^−ΔΔct^ method.

### Statistical analyses

Statistical analyses were conducted using GraphPad’s Prism 8 software (Version 8.3.1(332), https://www.graphpad.com). Two-way analysis of variance (ANOVA) was conducted for two-sample analyses and one-way analysis of variance (ANOVA) with post-hoc Tukey’s honest significant difference was conducted for multiple sample analyses.

## Supplementary Information

Supplementary Information.

## Data Availability

All data sets are included in the manuscript.
