# Supplementary material for: Anti-biofilm activity of caffeine against uropathogenic E. coli is mediated by curli biogenesis
Source: Sci Rep. 2022 Nov 7;12:18903. doi: 10.1038/s41598-022-23647-2 (PMC9640630; doi:10.1038/s41598-022-23647-2)
Supplement: Supplementary file 1 — Supplementary Information. [file 41598_2022_23647_MOESM1_ESM.pdf]

Anti-biofilm activity of caffeine against Uropathogenic *E. coli* is mediated by curli biogenesis.

Bhawna Rathi[1], Surbhi Gupta[1], Parveen Kumar[2], Veerbhan Kesarwani[3], Rakesh Singh Dhanda[4] , Sandeep Kumar Kushwaha[5], & Manisha Yadav[1,6*]

[1]Dr. B. R. Ambedkar Center for Biomedical Research, University of Delhi, New Delhi, India.

[2]Department of Urology, University of Alabama at Birmingham, Birmingham, AL, United States.

[3]Hap Biosolutions, Pvt. Ltd., Bhopal 462042, India

[4] Celluleris AB, VentureLab, Scheelevägen 15, 223 70 Lund, Sweden.

[5]Bioinformatics, DBT-National Institute of Animal Biotechnology(NIAB), Hyderabad, India

[6]Department of Clinical Sciences, Lund University, Malmö, Sweden.

***Corresponding Author**

Dr. Manisha Yadav, Professor

Dr. B. R. Ambedkar Centre for Biomedical Research

University of Delhi (North Campus)

Delhi-110007, INDIA

Phone: +91-11-27666241 Ext 241

E. mail: manisha.dhanda@gmail.com; manisha.yadav@med.lu.se

Short title – Curli-mediated *E.coli* biofilm inhibition by caffeine.

Supplementary Table S1:List of primers used for qRT-PCR

Gene	Primer	Sequence	Organism	Amplicon size
csg A	csg A-fwd<br>csg A-rev	CGGTAATGGTGCAGATGTTGG<br>TGAGTCACGTTGACGGAGGA	K12	192
csg A	csg A-fwd<br>csg A-rev	GGCCAAGGTTCTGATGACAGC<br>TTACCAAAGCCAACCTGAGTGA	CFT073	188
csg B	csg B-fwd<br>csg B-rev	TGCCAACGATGCCAGTATTTC<br>GTTGTGTCACGCGAATAGCC	K12, CFT073	158
csg C	csg C-fwd<br>csg C-rev	TATTACTCCTTGCGGCACTTTC<br>TCTGACTTTGCCCTGAACTGC	K12, CFT073	156
csg D	csg D-fwd<br>csg D-rev	CGTACCGCGACATTGAAAACT<br>GGGCTGATTCCGTGCTGTTA	K12, CFT073	183
csg E	csg E-fwd<br>csg E-rev	TGGCCATGATTTTTACCGAGC<br>CTGGCGACGATTTAGTGCTTC	K12, CFT073	223
csg F	csg F-fwd<br>csg F-rev	GCGTGTCAAACATGCAGTAGT<br>GGGCCTGAGCGCTATTTAAT	K12, CFT073	140
csg G	csg G-fwd<br>csg G-rev	CGAATCCCGCTGCAATCTTT<br>GGATCTCGCCGGTACTCACAT	K12, CFT073	187
16s rRNA [1]	16s rRNA-fwd<br>16s rRNA-rev	GCTCGTGTTGTGAAATGTTGGG<br>CTTATGAGGTCCGCTTGCT	K12, CFT073	222

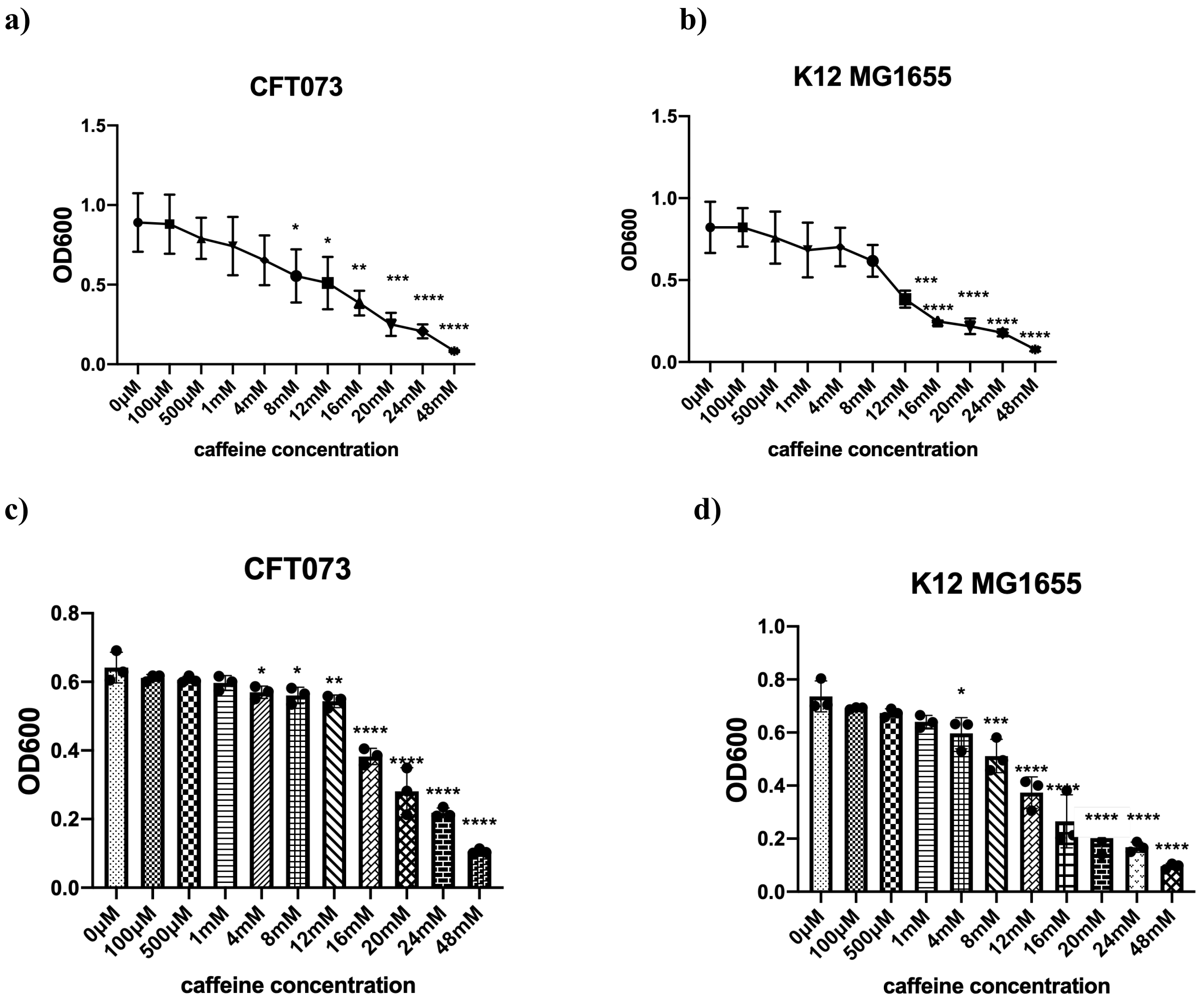

Supplementary Figure S1. Effect of caffeine on CFT073 and K12 MG1655 cells and their biofilms: a) MIC determination of caffeine against CFT073. Similar number of CFT073 cells($1*10^7$)were allowed to grow in the presence of various concentrations of caffeine for 24 hrs and optical density was measured at 600nm b) MIC determination of caffeine against K12 MG1655.c) MBIC determination of caffeine against CFT073. Biofilms were allowed to form in the presence of various concentrations of caffeine for 48 hrs and optical density was measured at 600nm d) MBIC determination of caffeine against K12 MG1655. Data represent an average of three independent experiments presented as Mean ± SD where * $p<0.05$, **$p<0.01$, ***$p<0.001$, and ****$p<0.0001$ indicates a statistically significant difference relative to the untreated control (one-way ANOVA).

a **b**

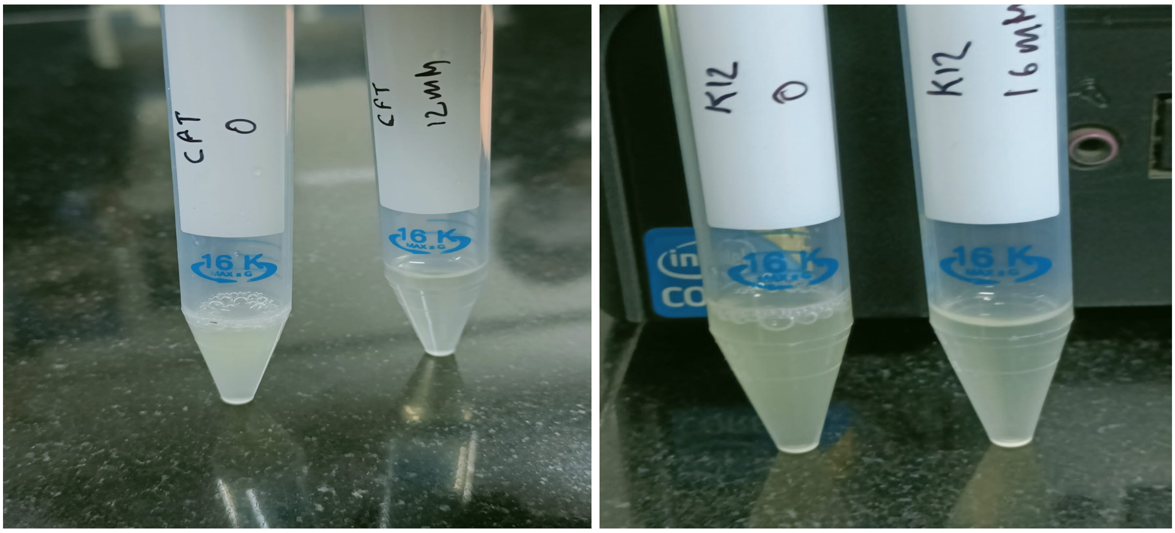

Supplementary Figure S2. Photographic images. Determination of MIC of caffeine against a) CFT073 inoculated without and with 12mM caffeine; b) K12 MG1655 inoculated without and with 16mM caffeine.

a **b**

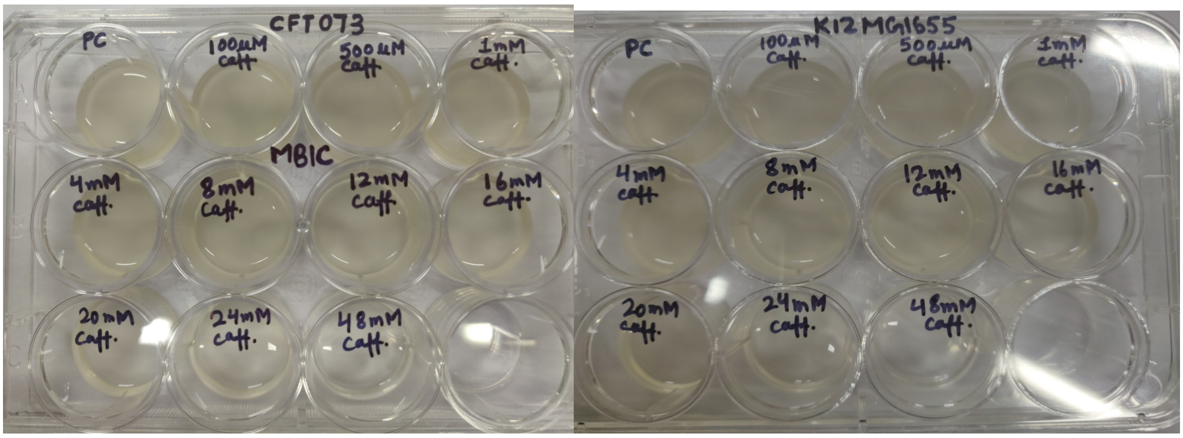

Supplementary Figure S3. Photographic images. Determination of MBIC of caffeine against CFT073 and K12 MG1655 a) CFT073 biofilm with different concentrations of caffeine and b) K12 MG1655 biofilm with different concentrations of caffeine.
